# Primary periocular basal cell carcinoma: recurrence, risk factors and follow-up^؆^

**DOI:** 10.1016/j.abd.2026.501334

**Published:** 2026-04-20

**Authors:** Raquel Galvão Bezerra, Daniela Cristina dos Santos Souza, José Eduardo Correntes, Roberta Lilian Fernandes Sousa, Silvana Artioli Schellini

**Affiliations:** a Department of Surgical Specialties and Anesthesiology, Faculty of Medicine, Universidade Estadual Paulista, Botucatu, SP, Brazil; b Department of Pathology, Faculy of Medicine, Universidade Estadual Paulista, Botucatu, SP, Brazil; c Department of Biodiversity and Biostatistics, Instituto de Biociências, Universidade Estadual Paulista, Botucatu, SP, Brazil

Dear Editor,

Primary basal cell carcinoma (BCC) is the most common periocular malignant tumor.^1^ Periocular tumors present therapeutic challenges due to the need for radical surgical excision while preserving eyelid function and facial aesthetics.

Although established guidelines recommend long-term follow-up for patients diagnosed with BCC due to the high risk of developing new lesions,^1^ Brazil lacks a national registry for skin or periocular cancers. Available data are derived from small hospital-based studies, and national guidelines do not include standardized long-term follow-up protocols, leaving monitoring decisions to individual clinicians.

To propose a tailored follow-up strategy for the Brazilian context, we retrospectively reviewed patients with primary periocular BCC who underwent surgical excision at our university hospital between March 2012 and December 2019 (Ethics approval: 39820420.1.0000.5411). All included patients who had complete excision of primary periocular BCC, confirmed histological subtype, documented margin status, and at least 12-months of follow-up. Non-periocular BCCs, recurrent tumors, prior radiotherapy or cryotherapy, immunosuppression, Gorlin-Goltz syndrome, and incomplete records were excluded.

We analyzed 135 histologically confirmed periocular BCC lesions from 116 patients, averaging 19.28 lesions treated annually. Periocular BCC predominantly affected elderly individuals, with a mean age of 69.47 ± 12.16 years old (range: 37–93) and a median age of 72. This trend likely reflects cumulative sun exposure, reduced DNA repair capacity, and age-related decline in immune response.^2^

Of the patients, 63 (54.31%) were female, with no significant gender difference in the occurrence of BCC. A higher incidence of periocular BCC in women has already been reported.^3^ The vast majority of patients (111/95.69%) had phototypes I or II, reinforcing the established link between BCC and lighter pigmentation, especially in individuals chronically exposed to sunlight. In contrast, darker skin provides greater UV protection and is associated with lower BCC risk.^4^

The mean tumor size was 7.23 ± 4.96 mm (range: 2–40 mm; median: 8 mm). The lower eyelid was the most affected site (110/81.48%), followed by the medial canthus (11/8.15%), upper eyelid (7/5.19%), lateral canthus (6/4.44%), and eyebrow (1/0.74%), consistent with previous studies.^5^, ^6^ The lower eyelid and medial canthus are more exposed to UV radiation and inflammatory agents such as tear components. The upper eyelid, protected by the brow and orbital anatomy, showed a lower frequency. No significant laterality effect was noted, although some studies suggest a higher incidence on the side of the face more exposed during driving.^7^

Tumors were generally conventionally excised under local anesthesia with 2–5 mm safety margins, depending on lesion size, consistent with other reports.^8^ Wider excision is recommended for aggressive subtypes of tumors (sclerodermiform, mixed), though it may compromise cosmetic and functional outcomes.^9^

In our clinic, frozen section or surgical excision with a safety margin is reserved for lesions with indistinct borders. This differs from other approaches that use frozen sections routinely or opt for wide local excision or Mohs surgery.^1^

The most common histological subtype was nodular (59/43.70%), followed by mixed (52/38.52%) (Table 1). Perineural invasion occurred in only one case (1/0.74%), consistent with other studies.^5^, ^6^, ^10^

**Table 1:** Histological classification and surgical margins of periocular BCC treated at Faculty of Medicine ‒ UNESP, 2025.

Variable	Frequency	%
*Histological classification*		
Nodular	59	43.70
Mixed	52	38.52
Sclerodermiform	7	5.19
Multifocal	2	1.48
Other^a^	2	1.48
No information	13	9.63
*Perineural involvement*		
No	130	96.3
Yes	1	0.74
No information	4	2.96
*Committed margins*		
No	110	81.48
Yes	14	10.37
No information	3	2.22
*Committed margins and location*^b^		
Deep	11	42.31
Lateral	10	38.46
Nasal	2	7.69
Upper	2	7.69
Lower	1	3.85

a Others: Micronodular, multifocal, infiltrative, fibroepithelial, basosquamous, plexiform, keratotic and metatypy (WHO, 2006).

b More than one margin can be affected (Total = 26).

We observed nine recurrences: 6 (66.67%) occurred in lesions with clear margins, underscoring that negative margins do not eliminate recurrence risk; 3 (33.33%) recurrences were from lesions with compromised margins. Of the 14 cases with involved margins, only 3 (21.43%) recurred. Reported recurrence rates for positive margins vary widely,^5^, ^8^, ^9^ depending on histological subtype, surgical excision type, and follow-up duration.^1^

Recurrences in our cohort were significantly associated with histological subtype (p = 0.037), being more frequent in nodular (3/33.33%), mixed (2/22.22%), and sclerodermiform (2/22.22%) subtypes. While nodular BCC is generally well-defined and amenable to complete excision, its recurrence rate in our sample likely reflects its high prevalence rather than intrinsic aggressiveness. In mixed BCC, the lesion may be composed of either high-risk or low-risk subtypes, making it important for the histological report to specify which subtypes are present in the lesion. Conversely, aggressive subtypes such as sclerodermiform are known to pose greater risks of incomplete excision and recurrence. Most recurrences occurred in the lower eyelid (8/88.89%) or in the medial canthus (1/11.11%), which were also the most common tumor locations. No significant association was found between recurrence and tumor size or margin status. Tumor-free surgical margins were achieved in 110 (81.48%) lesions, while compromised margins were identified in 14 (10.37%) cases. Twelve (8.89%) cases had indefinite margins; a known possibility related to the quality of some specimens.

The most frequently affected margin was the deep plane (11/42.31%), like other reports.^1^ Although BCC typically exhibits limited depth invasion, meticulous excision of the deep portion remains essential. In the medial canthus, erroneous efforts to preserve the lacrimal drainage system may inadvertently result in incomplete excision. Nevertheless, complete removal must remain the surgical goal to prevent orbital invasion and associated morbidity.

The mean follow-up duration was 3.41 ± 2.68 years (median: 2.84). Most patients (88/75.86%) remained in follow-up. Thirty-nine (33.62%) were lost to follow-up after two years, and 4 (3.45%) died from unrelated causes. No BCC metastases were reported. Table 2 presents recurrence according to histological subtype, committed margins and time to observe lesion recurrence. Mean time to recurrence was 2.92 ± 2.37 years (range: 1.09–6.25; median: 1.39-years).

**Table 2:** Recurrence after exeresis according to histological subtype and years.

Histological subtype	Committed margins	Reccurrence (years)
Mixed	No	1,23
No information	No	1,68
Mixed	Yes	1,39
Sclerodermiform	No	1,09
Others	Yes	1,34
Nodular	Yes	1,34
Sclerodermiform	No	6,64
Nodular	No	6,25
Nodular	No	5,18

According to other studies, periocular BCC recurrence rates are influenced by tumor location, size, histological subtype (more aggressive subtypes include micronodular, infiltrative, sclerosing, and morphea), margin status, immune function, systemic diseases, prior recurrences, and adjuvant treatments.^6^, ^10^

The approach to incomplete excision of BCC remains controversial. Since not all cases of low-risk tumors will recur,^9^ we recommend that re-excision should be evaluated on a case-by-case basis, considering factors such as high-risk anatomical location, involvement of deep or lateral margins, and the patient’s ability to adhere to follow-up.

Histologically compromised margins or incomplete lesion removal are not definitive predictors of recurrence. Recurrence may not occur even after several years, and clinical observation may be appropriate. Possible explanations for the absence of recurrence include devitalization of residual tumor cells by postoperative inflammation, containment within excision margins, differences in tumor biology, surgical technique, clinical management, or other biological mechanisms not yet fully understood. However, although re-excision is not necessary in all cases, it is strongly recommended for positive margins, particularly in high-risk histological subtypes, with meticulous excision of the affected margin.

Postoperative follow-up of excised periocular BCCs represents a significant workload for clinicians, and institutional protocols vary. For completely excised nodular BCC, recurrence risk is low, and follow-up for at least one year is recommended.

For aggressive subtypes, follow-up should extend to a minimum of five years, while recurrent tumors require indefinite surveillance. Approximately 30% of recurrences occur within the first year, and up to 82% within five years, reinforcing this period as the critical window for monitoring.

However, the purpose of follow-up is not only to detect local recurrence but also to identify new BCCs. Individuals who have already had one carry a 30%–40% risk of developing another,^9^ and therefore the patient requires close surveillance.

## Limitations and strengths

The limitations of this study include its retrospective design, which inherently involves incomplete information in medical records, the involvement of multiple surgeons, and histopathological evaluations performed by doctors in training ‒ factors that may affect the external validity and reproducibility of the findings. Another study limitation is that surgical margin assessment was not specified in 100% of the margins; such a comprehensive evaluation could have provided additional data to strengthen the conclusions. Notable strengths include the relatively large number of lesions analyzed, which contributes to the statistical robustness of the data. Additionally, all surgical procedures and histopathological evaluations were conducted under the supervision of a senior ophthalmologist and a dermatopathologist, enhancing diagnostic accuracy and classification reliability.

## Recommendations

• Early diagnosis, timely treatment, and consistent follow-up of periocular BCC are essential to reduce morbidity and mortality.
• Because patients with a history of BCC have an increased risk of developing additional tumors, follow-up should monitor both recurrence and the emergence of new lesions.
• Patient education on self-surveillance and sun protection is critical for early detection and prevention.
• Complete excision of low-risk lesions carries a low recurrence risk; such patients may be monitored for one year.
• Recurrences occurred on average three years post-surgery, with earlier recurrences (around one year) often associated with positive margins. Patients with compromised margins or confirmed recurrence should remain under annual follow-up, including routine dermatological assessments, for at least five years, regardless of subtype. After five years, patients may transition to primary care follow-up, provided that primary care teams are trained in lesion surveillance.

## ORCID ID

Raquel Galvão Bezerra: 0000-0002-1935-7451

Daniela Cristina dos Santos Souza: 0000-0002-8008-988X

José Eduardo Correntes: 0000-0001-5478-4996

Roberta Lilian Fernandes Sousa: 0000-0003-2872-2665

## Financial support

None declared.

## Authors' contributions

Raquel Galvão Bezerra: Collection, analysis, and interpretation of data; drafting and editing of the manuscript; approval of the final version of the manuscript.

Daniela Cristina dos Santos Souza: Analysis of histological slides; Approval of the final version of the manuscript.

José Eduardo Correntes: Statistical analysis; Approval of the final version of the manuscript.

Roberta Lilian Fernandes Sousa Meneghim: Analysis and interpretation of data; approval of the final version of the manuscript.

Silvana Artioli Schellini: Design and planning of the study; critical review of important intellectual content; approval of the final version of the manuscript.

## Research data availability

The entire dataset supporting the results of this study was published in this article.

## Conflicts of interest

None declared.
